# Hypoxia gradually augments metabolic and thermoperceptual responsiveness to repeated whole‐body cold stress in humans

**DOI:** 10.1113/EP089070

**Published:** 2020-11-16

**Authors:** Michail E. Keramidas, Roger Kölegård, Ola Eiken

**Affiliations:** ^1^ Division of Environmental Physiology Swedish Aerospace Physiology Center KTH Royal Institute of Technology Stockholm Sweden

**Keywords:** altitude, fatigue, hypothermia, immersion, sensitization, shivering, thermogenesis

## Abstract

**New Findings:**

- **What is the central question of this study?**
  In male lowlanders, does hypoxia modulate thermoregulatory effector responses during repeated whole‐body cold stress encountered in a single day?
- **What is the main finding and its importance?**
  A ∼10 h sustained exposure to hypoxia appears to mediate a gradual upregulation of endogenous heat production, preventing the progressive hypothermic response prompted by serial cold stimuli. Also, hypoxia progressively degrades mood, and compounds the perceived thermal discomfort, and sensations of fatigue and coldness.

**Abstract:**

We examined whether hypoxia would modulate thermoeffector responses during repeated cold stress encountered in a single day. Eleven men completed two ∼10 h sessions, while breathing, in normobaria, either normoxia or hypoxia ($P_{O_{2}}$: 12 kPa). During each session, subjects underwent sequentially three 120 min immersions to the chest in 20°C water (CWI), interspersed by 120 min rewarming. In normoxia, the final drop in rectal temperature (*T*_rec_) was greater in the third (∼1.2°C) than in the first and second (∼0.9°C) CWIs (*P* < 0.05). The first hypoxic CWI augmented the *T*_rec_ fall (∼1.2°C; *P* = 0.002), but the drop in *T*_rec_ did not vary between the three hypoxic CWIs (*P* = 0.99). In normoxia, the metabolic heat production ($\dot{M}$) was greater during the first half of the third CWI than during the corresponding part of the first CWI (*P* = 0.02); yet the difference was blunted during the second half of the CWIs (*P* = 0.89). In hypoxia, by contrast, the increase in $\dot{M}$ was augmented by ∼25% throughout the third CWI (*P* < 0.01). Regardless of the breathing condition, the cold‐induced elevation in mean arterial pressure was blunted in the second and third CWI (*P* < 0.05). Hypoxia aggravated the sensation of coldness (*P* = 0.05) and thermal discomfort (*P* = 0.04) during the second half of the third CWI. The present findings therefore demonstrate that prolonged hypoxia mediates, in a gradual manner, metabolic and thermoperceptual sensitization to repeated cold stress.

## Graphical abstract

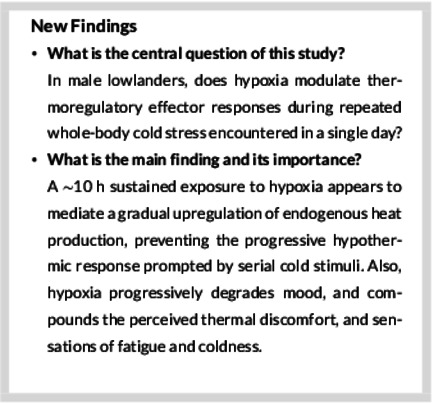

## 1 INTRODUCTION

In a thermally stressful environment, thermal homeostasis is preserved via the seamless recruitment of certain thermoeffectors. Particularly during cold exposure, peripheral vasoconstriction attenuates heat loss, muscular shivering enhances heat production and conscious thermo‐behavioural actions motivated by perceived thermal discomfort promote heat balance. Inefficiency, or perhaps failure, of these thermoregulatory adjustments to maintain thermal stability results in hypothermia.

When the potential to thermoregulate behaviourally is limited, survival time during cold exposure is largely dependent on shivering endurance; viz. the capacity to sustain, for an extensive period of time, a sufficient thermogenic rate capable of counterbalancing the rate of heat loss (Tikuisis, 1995). In support of anecdotal (Pugh, 1964, 1966) and case‐based (Bell, Tikuisis, & Jacobs, 1992; Thompson & Hayward, 1996) observations, two laboratory studies have suggested that, during protracted exposure to a high heat‐loss environment (e.g. cold‐water immersion), shivering‐active large muscle groups might exhibit signs of fatigue, leading to a progressive attenuation of metabolic heat production, and hence to a predisposition to hypothermia (Castellani, Young, Sawka, & Pandolf, 1998; Tikuisis, Eyolfson, Xu, & Giesbrecht, 2002). Firstly, Castellani et al. (1998) compared the thermoregulatory responses to three 2 h, 20°C water immersions repeated sequentially within a day, with the responses evoked by single immersions conducted in three separate days. The authors found that the serial immersions gradually blunted the thermogenic response to cold, because of a delay in the shivering onset threshold. Tikuisis et al. (2002) have also determined that the overall shivering intensity may remain constant in the later stages of lengthy cold‐water immersion, but the shivering sensitivity to falling deep‐body temperature appears to be diminished. Although in the aforementioned studies such metabolic downregulation may reflect a condition of ‘shivering fatigue’, it is plausible that it instead alludes to a rapid development of cold habituation (Tipton et al., 2013; Young, 1996), especially considering that, along with the thermogenic alterations, the sensation of coldness was alleviated as well (Castellani et al., 1998). Of interest in this regard is also the finding that, during protracted mild cold exposure, the shivering intensity appears to be preserved through a modification in fuel selection (i.e. carbohydrate and lipid oxidation decreases and increases, respectively) (Haman et al., 2016).

At high altitudes, hypoxia typically co‐exists with low ambient temperatures, and the risk of developing accidental hypothermia seems to be enhanced, especially when heat loss is aggravated by rain, snow and/or wind (Ainslie & Reilly, 2003; Procter, Brugger, & Burtscher, 2018). Human‐based studies have yielded evidence that systemic hypoxia may impinge on cold defence mechanisms, jeopardizing the capacity to maintain thermal balance (for review, see Mugele, Oliver, Gagnon, & Lawley, 2020). Thus, superimposition of acute hypoxia on short‐duration cold stress may blunt peripheral vasoconstriction (Cipriano & Goldman, 1975; Johnston, White, Wu, Bristow, & Giesbrecht, 1996; Keramidas, Kolegard, Mekjavic, & Eiken, 2019; Simmons, Fieger, Minson, & Halliwill, 2010) and/or suppress shivering thermogenesis (Johnston et al., 1996; Keramidas et al., 2019; Kottke, Phalen, Taylor, Visscher, & Evans, 1948), accelerating the body core cooling; whereas thermal perception and behaviour may remain intact (Golja & Mekjavic, 2003; Golja, Kacin, Tipton, & Mekjavic, 2005). During acute exposures, the impact of hypoxia on cold‐defence thermoregulation appears to be dose dependent, i.e. not only the degree, but also the duration of hypoxic stimulus may affect the interactive responses (cf. Simmons et al., 2010; Simmons, Barrett‐O'Keefe, Minson, & Halliwill, 2011). Information is lacking, however, for whether adaptive mechanisms may modify these interactions during prolonged or iterative exposures to hypoxia and cold.

The purpose of the present study therefore was to examine whether, and to what extent, hypoxia would modulate thermoregulatory effector responses during continual exposures to moderate cold. To address this question, thermal, cardiovascular and perceptual responses were monitored during 2 h cold water immersions repeated three times serially in a 10 h period, while subjects were breathing, in normobaria, either normoxia or hypoxia. Although it might be argued that sustained exposure to cold air would have increased the ecological validity of the study, we utilized 20°C water immersion (i.e. a moderate‐intensity cold stimulus) to elicit, in a safe manner and in a relatively short period of time, sufficient degrees of deep‐body cooling to stimulate moderate levels of shivering (Castellani et al., 1998). We hypothesized that the ∼10 h hypoxic stressor would attenuate the cold‐induced thermogenesis and peripheral vasoconstriction, and thus aggravate the progressive reduction in body core temperature prompted by the repeated cold stimuli.

## 2 METHODS

### 2.1 Ethical approval

The experimental protocol was approved by the Human Ethics Committee of Stockholm (2018/1433‐31), and conformed to the standards set by the *Declaration of Helsinki*, except for registration in a database. Subjects were informed in detail about the experimental procedures before giving their written consent to participate, and were aware that they could terminate their participation at any time.

### 2.2 Subjects

Based on the mean effect sizes for changes in body core temperature from a previous study using a similar experimental protocol (Castellani et al., 1998), a minimum sample size of 10 individuals was required to determine significant differences (α = 0.05, β = 0.8; G*Power 3.1 software, Heinrich‐Heine‐Universität, Dusseldorf, Germany; Faul, Erdfelder, Lang, & Buchner, 2007). Eleven healthy male lowlanders therefore participated in the study: mean (range): age 26 (23–31) years; body mass 83.0 (77.3–90.1) kg; height 182 (171–188) cm; body surface area 2.05 (1.91–2.16) m^2^; total skinfold thickness 74.6 (52.0–108.2) mm; body fat 10.3 (6.9–15.1)%; peak oxygen uptake (${\dot{V}}_{O_{2}\mathrm{peak}}$) in absolute values 3.7 (2.7–4.4) l min^−1^; ${\dot{V}}_{O_{2}\mathrm{peak}}$ in relative values 44.9 (33.7–51.4) ml min^−1^ kg^−1^; estimated peak shivering metabolic rate (Shiv_peak_) 17.4 (14.5–19.7) ml min^−1^ kg^−1^, which was calculated by the equation: $\mathrm{Shi}v_{\mathrm{peak}}=30.5+(0.348\times {\dot{V}}_{O_{2}\mathrm{peak}}-\left(0.909\times \mathrm{BMI}\right)-\left(0.233\times \mathrm{age}\right)$ (Eyolfson, Tikuisis, Xu, Weseen, & Giesbrecht, 2001). Subjects were nonsmokers, had no history of any cold injury, and were not taking any medication. None of them were regularly exposed to cold water, or had sojourned at altitude ≥500 m during the month preceding the experiments. Subjects were instructed to: (i) abstain from alcohol and strenuous exercise for 24 h prior to each test session, (ii) refrain from caffeine during the testing day, and (iii) maintain their habitual sleep (≥7 h) and eating routines the day before the test sessions.

### 2.3 Study design

The study was performed between November and February in a laboratory of the Division of Environmental Physiology, Royal Institute of Technology (Solna, Sweden). The study employed a single‐blinded, repeated‐measures design. Subjects visited the laboratory on three separated occasions: visit 1 was the preliminary session, and visits 2 and 3 were the main ∼11 h test sessions. During the test sessions, subjects, who were breathing continually via an oronasal mask either a normoxic (fraction of O_2_ ($F_{O_{2}}$): 0.21; partial pressure of oxygen ($P_{O_{2}}$): 21 kPa) or a hypoxic ($F_{O_{2}}$: 0.12, $P_{O_{2}}$: 12 kPa, ambient simulated elevation of ∼4300 m; cf. West, 1996) gas, underwent three 120 min cold‐water (20°C) immersions (CWIs; 1st CWI: CWI_A_, 2nd CWI: CWI_B_, 3rd CWI: CWI_C_), interspersed by a 120 min rewarming period. The use of hypoxia at 0.12 $F_{O_{2}}$ was based on previous work showing that the cold‐defence thermoeffectors are attenuated during acute exposure to such hypoxic stimulus (Johnston et al., 1996; Keramidas et al., 2019). CWI_A_, CWI_B_ and CWI_C_ were performed at approximately 08.00, 12.00 and 16.00 h, respectively. For the individual subject, the normoxic and hypoxic test sessions were performed within a 4 week period, and were separated by at least a week. Subjects were asked to avoid cold exposures, and to maintain their daily exercise and dietary routines during the intervening period. The order of the sessions was alternated among subjects: the first session was normoxic for five subjects and hypoxic for six subjects. Testing was conducted by the same investigators.

#### 2.3.1 Preliminary session

Approximately a week prior to the main test session, subjects were thoroughly familiarized with the equipment and experimental procedures. Subjects’ ${\dot{V}}_{O_{2}\mathrm{peak}}$ was determined by an incremental (25 W min^−1^) cycling trial to exhaustion. Body mass (accuracy 0.01 kg) was recorded with an electronic scale (Vetek, Väddö, Sweden). The body surface area was derived from measures of body mass and height (Du Bois & Du Bois, 1916). Skinfold thicknesses were measured with a skinfold caliper (Harpenden, West Sussex, UK) at seven right‐side locations: triceps, subscapular, chest, suprailiac, abdominal, front thigh and midaxillary. Percentage body fat was calculated according to the equation of Jackson and Pollock (1978).

#### 2.3.2 Main test sessions

During the test sessions, the environmental conditions in the laboratory were kept constant: the mean (standard deviation; SD) temperature, relative humidity and barometric pressure were 27.1 (0.3)°C, 27 (6)% and 764 (12) mmHg, respectively. Subjects reported to the laboratory at 07.00 h following a 10 h fast, and became accustomed to the laboratory conditions for ∼40 min. During this period, they ate a standardized snack (snack 1), and completed two questionnaires (see below for details). After emptying their bowel and bladder, body mass was recorded, and the instrumentation was conducted. Subjects then assumed a resting semi‐reclining position on a gurney placed next to the water‐tank; they were equipped with an oronasal mask, and inspired either the normoxic or the hypoxic gas. The facemask was maintained throughout the session; it was removed temporarily during the two ∼5–15 min snack periods taking place after CWI_A_ (snack 2) and CWI_B_ (snack 3). Each CWI commenced with a 20 min baseline, while subjects rested on the gurney. Thereafter, subjects, who were always clad in non‐insulated swim pants, were immersed to the level of the xiphoid process in 20°C water for up to 120 min, or until the rectal temperature (*T*_rec_) dropped below 35°C. The water was continuously stirred, and its temperature was monitored by two thermistors (PT100, Texas Instrument, Dallas, USA) placed >10 cm from the body at different water depths; if necessary, the water temperature was adjusted by adding cold water from the tap. During all CWIs, subjects remained in a semi‐upright sitting position with their arms being supported at the level of the heart, above the water surface. Subjects watched movies throughout. After the completion of CWI_A_ and CWI_B_, subjects were removed from the cold‐water tank, dried with a towel, and a 120 min rewarming phase ensued, during which *T*_rec_ returned to the values of the CWI_A_ baseline. The rewarming phase consisted of a 30 min passive rewarming on the gurney (subjects lay in a sleeping bag equipped with an inner plastic lining), followed by active rewarming (immersion in ∼39.5°C water for ∼35 min), and again by passive rewarming until the next baseline phase. When needed, subjects urinated in an urinal bottle, while they were lying on the gurney during the rewarming; the amount of urinary output was not recorded. After the end of CWI_C_, subjects were towelled carefully, and their body mass was recorded.

In each snack period, subjects ate a 45 g protein bar (consisting of 10.9 g carbohydrates, 22.5 g protein and 4.3 g fat; Protein Pro chocolate, FCB, Lund, Sweden). Either in snack 2 or in snack 3, subjects were allowed to also have a 50 g cereal bar (consisting of 58 g carbohydrates, 4.6 g protein and 23.4 g fat; Corny Big, Schwartauer Werke, Bad Schwartau, Germany), or a cup of warm chocolate (consisting of a 28 g chocolate powder (18 g carbohydrates, 6 g protein and 0.4 g fat) in ∼200 ml water; O'boy; Mondelez, Upplands Väsby, Sweden). For each individual subject, the type and timing of snacks were identical in the two test sessions; the total energy intake was 665 (42) kcal per session. Snack 2 and snack 3 always took place immediately after the active rewarming; thus, subjects were reinstated to hypoxia for, at least, a 45 min period prior to the initiation of the subsequent CWI. During the snack time, subjects were allowed to drink water *ad libitum*; the total amount of consumed water did not differ between sessions (Normoxia: 616 (207) ml, Hypoxia: 741 (218) ml; *P* = 0.10).

### 2.4 Measurements

#### 2.4.1 Thermometry

*T*_rec_ was monitored continuously with a rectal thermistor (Yellow Springs Instruments, Yellow Springs, OH, USA) placed in a protective sheath and inserted 10 cm beyond the anal sphincter. Mean skin temperature (*T*_sk_) was derived from the unweighted average of skin temperatures, recorded with copper–constantan (T‐type) thermocouple probes (Physitemp Instruments Inc., Clifton, NJ, USA) at the left side of the forehead, upper arm, upper back, forearm, ring finger, chest, abdomen, thigh, calf and foot. All temperatures were sampled at 1 Hz with a NI USB‐6215 data acquisition system, and processed with LabVIEW software (version 2017, National Instruments, Austin, TX, USA). Prior to each test session, all temperature probes were calibrated against a certified reference thermometer (Ellab, Copenhagen, Denmark).

#### 2.4.2 Respiratory measurements

Throughout the test sessions, subjects breathed through a low resistance two‐way respiratory valve (model 2, 700 T‐Shape, Hans Rudolph, Shawnee, KS, USA). The inspiratory side of the valve was connected via respiratory corrugated tubing to a bag filled with the premixed humidified breathing gas. Inspired and expired gases were sampled continuously, from either side of the respiratory valve. Oxygen uptake (${\dot{V}}_{O_{2}}$), carbon dioxide production (${\dot{V}}_{CO_{2}}$), respiratory exchange ratio (RER), expired ventilation (${\dot{V}}_{E}$), energy expenditure (EE), tidal volume (*V*_T_), respiratory frequency (*f*_R_) and partial pressure of end‐tidal carbon dioxide ($P_{\mathrm{ETC}O_{2}}$) were measured breath‐by‐breath using a metabolic unit (Quark PFT; Cosmed, Rome, Italy). In accordance with the manufacturer's recommendation, a two‐point calibration was performed (room air: 20.93% O_2_ and 0.03 CO_2_, and certified gas mixture: 16.00% O_2_–5.00% CO_2_) before each session (Simpson, Debevec, Eiken, Mekjavic, & Macdonald, 2016). The pneumotachograph was calibrated before each session with a 3 litre syringe.

#### 2.4.3 Arterial pressures, heart rate and capillary oxyhaemoglobin saturation

During the baseline and CWIs, beat‐to‐beat systolic (SAP), diastolic (DAP) and mean (MAP) arterial pressures were measured continuously using a volume‐clamp technique (Finometer, Finapres Medical Systems BV, Amsterdam, the Netherlands), with the pressure cuff placed around the middle phalanx of the left middle finger, and with the reference pressure transducer positioned at the level of the heart. The Finometer‐derived values were verified intermittently by electro‐sphygmomanometry (Omron, M6, Kyoto, Japan). Heart rate (HR) was derived from the arterial pressure curves as the inverse of the inter‐beat interval. Capillary oxyhaemoglobin saturation ($S_{pO_{2}}$) was monitored at 5 min intervals with an earlobe pulse oximeter (Radical‐7, Masimo, Irvine, CA, USA).

#### 2.4.4 Skin blood flow

Local skin blood flow was monitored at a rate of 1 Hz on the dorsal side of the left forearm (non‐glabrous skin location), and the palmar side of the distal phalanx of the left index finger (glabrous skin location) by laser‐Doppler flowmetry (VMS‐LDF2; Moor Instruments, Axminster, UK) using optic probes (VP1/7; Moor Instruments), which were firmly connected to the skin with double‐sided adhesive tape. All laser‐Doppler probes were calibrated before each session against Brownian motion with a standardized colloidal suspension of polystyrene microspheres. Skin blood flow was reported as cutaneous vascular conductance (CVC), calculated as skin blood flow divided by MAP.

#### 2.4.5 Arterial blood flow

During each baseline phase, and at minutes 30, 60, 90 and 120 of the CWIs, flow in the right radial, ulnar and brachial arteries was estimated by measurements of mean flow velocity and diameter using ultrasonographic equipment (Siemens Acuson S2000, Siemens Healthcare AB, Stockholm, Sweden), with a linear array multifrequency transducer (4–9 MHz). Two‐dimensional measurements of the arterial lumen were made from B‐mode images in longitudinal view during end‐diastole. Flow velocities were collected with the sample volume covering about 100% of the arterial lumen, and with an angle correction always kept at 50 deg and parallel to the vessel walls. Arterial flow was calculated by multiplying the arterial lumen cross‐sectional area by the time integral of the mean flow velocity over a period of three to five cardiac cycles. The brachial artery was insonated ∼5 cm proximal to the cubital fossa, and the radial and ulnar arteries ∼5 cm proximal to the wrist. To ensure that insonation of the same segment of the vessel was performed in subsequent measurements and experiments, intra‐ and extra‐vascular landmarks were chosen.

#### 2.4.6 Capillary glucose

During each baseline phase and at minute 95 of the CWIs, capillary blood was sampled from the right finger to measure glucose concentration. The skin was punctured with a lancet (Accu‐Check, Scoftclix, Basel, Switzerland), and the second drop of blood was placed on a strip and immediately analysed with a portable analyser (Accu‐Chek, Aviva, Roche, Manheim, Germany).

#### 2.4.7 Perceptual measurements

During each baseline, and at minutes 1, 30, 60, 90 and 120 of the CWIs, subjects were asked to provide ratings of whole‐body thermal sensation (from 1, cold, to 7, hot) and thermal comfort (from 1, comfortable, to 4, very uncomfortable). At the same time intervals, the affective valence was also assessed by means of the feeling scale (from −5, very bad, to +5, very good).

Approximately 10 min before each baseline phase, and at minute 100 of the CWIs, subjects were requested to complete the following questionnaires, based on how they felt at that particular moment: (i) the Profile of Mood States–Short Form (Shacham, 1983), which is a 37‐item self‐evaluation questionnaire of six subscales: tension, depression, anger, vigour, fatigue and confusion. The description of subjects’ feelings was provided based on a five‐point scale with anchors from 0, not at all, to 4, extremely. (ii) the 2018 Lake Louise Score, which is a self‐assessment questionnaire of acute mountain sickness. The Lake Louise Score evaluates the severity of headache, nausea, dizziness and fatigue; each item is rated with a score of 0, no symptoms, to 3, severe symptoms. The presence of acute mountain sickness was defined by a value ≥3, including headache. Both questionnaires were presented in hardcopy format, and were explained to the subjects by the same investigator prior to each test session. Subjects replied to the questions within ∼3–5 min.

### 2.5 Data and statistical analyses

Only data collected during the baseline and CWI phases were analysed. Baseline values were calculated as averages of the final 10 min of the 20 min baseline phase. All physiological data obtained during CWI were reduced to 60 s averages. Due to the inter‐ and intra‐individual variability in the duration of the CWIs, data were expressed as a function of the absolute time completed by all subjects, including the corresponding final value obtained in each CWI. For selected variables, the averages of the last 60 min of each CWI (CWI_L‐60_) were also evaluated.

The individual shivering thresholds, indicated by a sustained elevation in ${\dot{V}}_{O_{2}}$ (≥100 ml min^−1^ increase from the initial plateau observed upon immersion), were derived from the responses of ${\dot{V}}_{O_{2}}$ relative to changes in *T*_rec_ (Δ*T*_rec_) (Arnold, Hemsley, Hodder, Havenith, & Lloyd, 2020; Mekjavic, Sundberg, & Linnarsson, 1991). Metabolic heat production ($\dot{M}$) was calculated by the equation: $\dot{M}$ = (0.23 × RER + 0.77) × 5.873${\dot{V}}_{O_{2}}$ × 60, and reported in watts (Gagge & Gonzalez, 1996). Linear regression analysis was performed over each CWI_L‐60_ to evaluate the individual sensitivity (slope) of the $\dot{M}$ response as a function of Δ*T*_rec_. Relative shivering intensity (%Shiv_peak_) was determined by dividing ${\dot{V}}_{O_{2}}$ obtained during CWI_L‐60_ by Shiv_peak_ (Eyolfson et al., 2001).

Normality of distribution for all datasets was assessed using the Shapiro–Wilk test. A two‐way (breathing condition (normoxia × hypoxia) × CWI trial (CWI_A_ × CWI_B_ × CWI_C_)) or three‐way (breathing condition × CWI trial × time) repeated measures analysis of variance (ANOVA) was employed for all physiological variables. Mauchly's test was conducted to assess the sphericity and, if necessary, the Greenhouse–Geiser ε correction was used to adjust the degrees of freedom. When ANOVA revealed significant effects, multiple pairwise comparisons were performed with Tukey's honestly significant difference *post hoc* test. Student's paired *t* test was used to determine differences in the amount of consumed water and the changes in body weight between the two test sessions. The duration of CWIs was analysed with Kaplan–Meier survival curves and log‐rank (Mantel–Cox χ^2^) test. Differences in perceptual responses were evaluated with Friedman's test, and the Wilcoxon signed‐rank test was used as a *post hoc* test. Effect sizes are reported as partial eta‐squared (η_p_^2^; values of ≤0.02, ≤0.13 and ≥0.26 are considered as small, moderate and large, respectively). For the parametric pairwise‐comparisons, effect sizes were calculated using Cohen's *d* (values of ≤0.2, ≤0.5 and ≥0.8 are considered as small, moderate and large, respectively). The internal consistency of the POMS‐SF subscales was examined with Cronbach's α coefficient. The relationship between Lake Louise Score values and Δ*T*_rec_, and $\dot{M}$ was assessed using a Spearman rank correlation coefficient (*r*_s_). Statistical analyses were conducted using Statistica 8.0 (StatSoft, Tulsa, OK, USA) or Prism 8.0 (GraphPad Software Inc., San Diego, CA, USA). Unless otherwise stated, data are presented as mean values with 95% confidence intervals (CI), which were calculated using a non‐central *t* distribution. The α level of significance was set *a priori* at 0.05.

## 3 RESULTS

Apart from in the normoxic CWI_A_, one or several subjects terminated the 120 min CWIs prematurely, because *T*_rec_ reached the critical temperature of 35°C (number of subjects who completed the 120 min CWIs: Normoxia: CWI_A_, 11; CWI_B_, 10; CWI_C_, 9; and Hypoxia: CWI_A_, 10; CWI_B_, 8; CWI_C_, 8). The duration of CWIs, however, was not significantly different (mean duration (range): Normoxia: CWI_A_, 120 min; CWI_B_, 117 (86–120) min; CWI_C_, 115 (90–120) min; Hypoxia: CWI_A_, 119 (105–120) min; CWI_B_, 116 (96–120) min; CWI_C_, 114 (76–120) min; χ^2^ = 4.83, *P* = 0.43).

$S_{pO_{2}}$ was consistently lower (*P* < 0.001) in hypoxia (78 (2)%) than in normoxia (98 (1)%). In hypoxia, symptoms of acute mountain sickness were determined in six subjects during CWI_B_, and in seven subjects during CWI_C_. The mean (range) values of the Lake Louise Score were: Normoxia: CWI_A_, 0 (0–3); CWI_B_, 1 (0–2); CWI_C_, 1 (0–3); Hypoxia: CWI_A_, 2 (0–3); CWI_B_, 2 (0–6); CWI_C_, 3 (0–6); *P* = 0.04.

Blood glucose concentration, which was similar across the baseline phases, was reduced (*P* ≤ 0.001) during all CWIs, and to a similar extent (Normoxia: Baseline CWI_A_, 5.8 (0.4) mmol l^−1^; CWI_A_, 5.0 (0.3) mmol l^−1^; Baseline CWI_B_, 6.1 (0.4) mmol l^−1^; CWI_B_, 4.8 (0.4) mmol l^−1^; Baseline CWI_C_, 6.1 (0.5) mmol l^−1^; CWI_C_, 4.7 (0.5) mmol l^−1^; Hypoxia: Baseline CWI_A_, 5.7 (0.4) mmol l^−1^; CWI_A_, 5.1 (0.3) mmol l^−1^; Baseline CWI_B_, 6.3 (0.6) mmol l^−1^; CWI_B_, 4.8 (0.6) mmol l^−1^; Baseline CWI_C_, 6.1 (0.6) mmol l^−1^; CWI_C_, 5.0 (0.4) mmol l^−1^; *P* = 0.29, η_p_^2^ = 0.11).

Body mass was reduced by both test sessions (*P* ≤ 0.001), with reductions of ∼1.5% in normoxia (Before: 83.0 (3.6) kg; After: 81.8 (3.4) kg) and ∼1.9% in hypoxia (Before: 82.9 (3.9) kg; After: 81.3 (3.9) kg); the changes were similar in the two sessions (*P* = 0.31, *d* = 0.16).

### 3.1 Thermal responses

**FIGURE 1:**
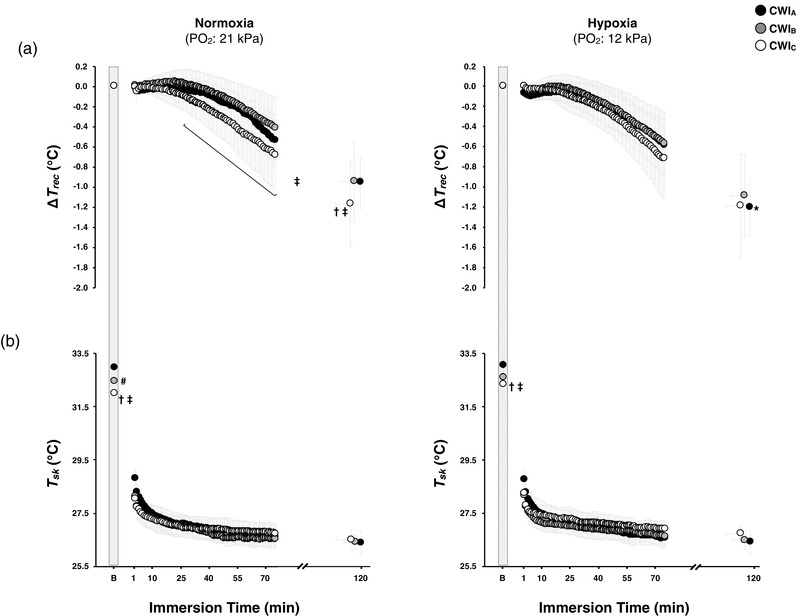
Time course of changes in rectal temperature relative to baseline values (Δ*T*_rec_; a), and skin temperature (*T*_sk_; b) during the three successive cold‐water (20°C) immersions (CWI_A_: 1st trial, CWI_B_: 2nd trial, CWI_C_: 3rd trial) performed in normoxia and hypoxia. Data are expressed as a function of the immersion time completed by all subjects, including the final value obtained in each CWI. B: baseline. Values are mean (95% CI). Data in all trials were significantly different from baseline (*P* < 0.001). Significant difference # between CWI_A_ and CWI_B_, † between CWI_A_ and CWI_C_, ‡ between CWI_B_ and CWI_C_, and * between the normoxic and hypoxic CWI_A_

The mean time series for Δ*T*_rec_ and *T*_sk_ are depicted in Figure 1. Baseline *T*_rec_ did not differ between trials (Normoxia: CWI_A_, 37.0 (0.2)°C; CWI_B_, 37.0 (0.1)°C; CWI_C_, 37.0 (0.1)°C; Hypoxia: CWI_A_, 37.0 (0.1)°C; CWI_B_, 37.0 (0.1)°C; CWI_C_, 37.1 (0.1)°C; *P* = 0.36, η_p_^2^ = 0.09). *T*_rec_ dropped during all CWIs (final value of *T*_rec_ in Normoxia: CWI_A_, 36.1 (0.3)°C; CWI_B_, 36.1 (0.4)°C; CWI_C_, 35.8 (0.4)°C; Hypoxia: CWI_A_, 35.8 (0.3)°C; CWI_B_, 35.8 (0.4)°C; CWI_C_, 35.9 (0.6)°C; *P* ≤ 0.001). In normoxia, the final Δ*T*_rec_ was similar in CWI_A_ and CWI_B_ (∼0.9°C; *P* = 0.95); but it was greater in CWI_C_ (∼1.2°C) than in CWI_A_ (*P* = 0.05, *d* = 0.39) and in CWI_B_ (*P* = 0.02, *d* = 0.35). Hypoxia augmented the fall in *T*_rec_ during CWI_A_ by ∼0.3°C (*P* = 0.002, *d* = 0.62). Yet the final Δ*T*_rec_ did not vary between the three hypoxic CWIs (*P* = 0.99).

In normoxia, the baseline *T*_sk_ was lower in CWI_B_ and CWI_C_ than in CWI_A_ (*P* ≤ 0.001), and in CWI_C_ than in CWI_B_ (*P* = 0.02). In hypoxia, the baseline *T*_sk_ was lower in CWI_B_ and CWI_C_ than in CWI_A_ (*P* ≤ 0.001). No differences were noted between the normoxic and hypoxic baseline *T*_sk_ (*P* ≥ 0.50). Overall, the cold‐induced reduction (*P* ≤ 0.001) in *T*_sk_ was similar in the six CWIs (*P* = 0.72, η_p_^2^ = 0.07). Yet, during the hypoxic CWI_L‐60_, *T*_sk_ was higher by ∼0.4°C (*P* = 0.05, *d* = 0.76) in CWI_C_ than in CWI_A_ (CWI_A_, 26.5 (0.4)°C; CWI_B_, 26.7 (0.3)°C; CWI_C_, 26.9 (0.3)°C). *T*_sk_ did not differ between the three normoxic CWI_L‐60_ (CWI_A_, 26.6 (0.4)°C; CWI_B_, 26.4 (0.3)°C; CWI_C_, 26.7 (0.4)°C; *P* > 0.05).

### 3.2 Cardiorespiratory responses

**FIGURE 2:**
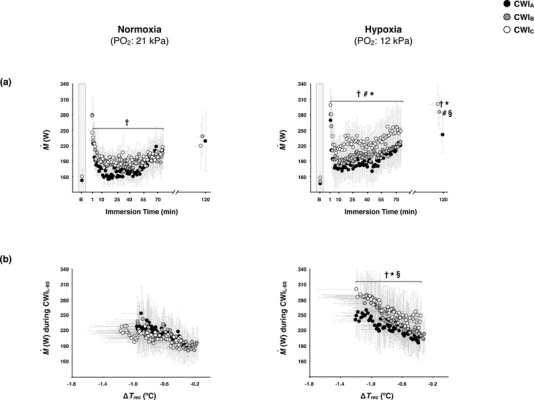
Time course of metabolic heat production ($\dot{M}$) during the total (a; data are expressed as a function of the immersion time completed by all subjects, including the final value obtained in each CWI), and the last 60 min (CWI_L‐60_; b) period of the three successive cold‐water (20°C) immersions (CWI_A_: 1st trial, CWI_B_: 2nd trial, CWI_C_: 3rd trial) performed in normoxia and hypoxia. B: baseline. Values are mean (95% CI). Data in all trials were significantly different from baseline (*P* ≤ 0.001). Significant difference # between CWI_A_ and CWI_B_, † between CWI_A_ and CWI_C_, § between the normoxic and hypoxic CWI_B_, and * between the normoxic and hypoxic CWI_C_

**FIGURE 3:**
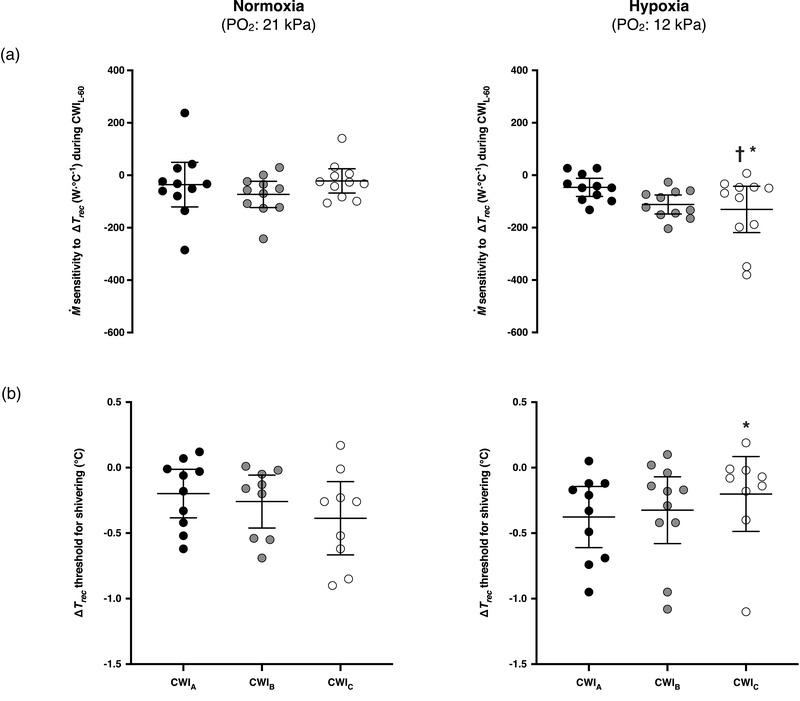
Mean (95% CI) and individual values of the metabolic heat production ($\dot{M}$) sensitivity (a) and shivering thresholds (b) obtained during the three successive cold‐water (20°C) immersions (CWI_A_: 1st trial, CWI_B_: 2nd trial, CWI_C_: 3rd trial) performed in normoxia and hypoxia. Number of subjects that exhibited a shivering response in normoxia: CWI_A_, 10; CWI_B_, 9; CWI_C_, 9; and in hypoxia: CWI_A_, 10; CWI_B_, 11; CWI_C_, 9. $\dot{M}$ sensitivity was calculated for the last 60 min of each immersion (CWI_L‐60_). Δ*T*_rec_: changes in rectal temperature relative to baseline values. Significant difference † between CWI_A_ and CWI_C_, and * between the normoxic and hypoxic CWI_C_

$\dot{M}$ did not vary across the baseline phases, and was enhanced (*P* ≤ 0.001) in all CWIs (Figure 2a). In normoxia, $\dot{M}$ was greater during the initial part of CWI_C_ than during the corresponding part of CWI_A_ (*P* = 0.02; Figure 2a); yet the difference was blunted during CWI_L‐60_ (*P* = 0.89; Figure 2b). The cold‐induced elevation in $\dot{M}$ was unaltered by hypoxia during CWI_A_ and CWI_B_ (∼15% from the hypoxic CWI_A_; *P* = 0.45), but was augmented during CWI_C_ (∼25% from the hypoxic CWI_A_; *P* ≤ 0.01; Figure 2a). The %Shiv_peak_ was similar in the normoxic CWIs, but was increased by hypoxia in CWI_B_ and CWI_C_ (*P* ≤ 0.01; Table 1). The $\dot{M}$ sensitivity to Δ*T*_rec_ was not modified by the repeated normoxic CWIs (*P* ≥ 0.80; Figure 3a), whereas it was gradually enhanced by the repeated hypoxic CWIs, and particularly prominent in the hypoxic CWI_C_ (*P* ≤ 0.05; Figure 3a). In eight out of eleven subjects, the shivering threshold was shifted towards a greater Δ*T*_rec_ in the hypoxic than the normoxic CWI_A_ (*P* = 0.13, *d* = 0.62). In comparison with the respective CWI_A_, the shivering onset (Figure 3b) was unaltered by the normoxic CWI_C_ (*P* = 0.07, *d* = 0.61), and by the hypoxic CWI_C_ (*P* = 0.10, *d* = 0.50). In the CWI_C_, however, the Δ*T*_rec_ threshold for shivering was lower in hypoxia than in normoxia (*P* = 0.03, *d* = 0.51).

**TABLE 1:** Mean (95% CI) cardiorespiratory responses obtained during the three successive cold‐water (20°C) immersions (CWI_A_: 1st trial, CWI_B_: 2nd trial, CWI_C_: 3rd trial) performed in normoxia and hypoxia

	Normoxia ($P_{O_{2}}$: 21 kPa)			Hypoxia ($P_{O_{2}}$: 12 kPa)		
	CWI_A_	CWI_B_	CWI_C_	CWI_A_	CWI_B_	CWI_C_
${\dot{V}}_{O_{2}}$ (l min^−1^)	0.60 (0.09)	0.63 (0.07)	0.62 (0.08)	0.64 (0.07)	0.73 (0.07)^*^	0.79 (0.09)^†‡*^
${\dot{V}}_{O_{2}}$ (l min^−1^) during CWI_L‐60_	0.64 (0.09)	0.62 (0.06)	0.62 (0.08)	0.67 (0.09)	0.72 (0.11)^*^	0.77 (0.11)^†*^
%Shiv_peak_ during CWI_L‐60_	45 (8)	43 (7)	44 (8)	47 (8)	51 (11)^*^	55 (12)^†*^
EE (kcal)	350 (52)	353 (41)	338 (38)	357 (38)	393 (43)	420 (57)^*^
${\dot{V}}_{CO_{2}}$ (l min^−1^)	0.50 (0.07)	0.52 (0.06)	0.49 (0.05)	0.53 (0.06)	0.58 (0.06)^*^	0.62 (0.07)^†‡*^
RER during CWI_L‐60_	0.81 (0.03)	0.79 (0.02)^#^	0.78 (0.03)^†^	0.83 (0.02)	0.80 (0.02)#	0.79 (0.02)^†^
${\dot{V}}_{E}$ (l min^−1^)	14.2 (1.9)	15.0 (2.3)	14.0 (1.4)	16.5 (2.0)^*^	19.9 (2.5)^#*^	21.5 (2.3)^†‡*^
*V*_T_ (l)	1.23 (0.17)	1.26 (0.14)	1.09 (0.13)	1.34 (0.20)^*^	1.48 (0.17)^*^	1.54 (0.25)^*^
*f*_R_ (breaths min^−1^)	12 (2)	13 (3)	14 (2)	13 (3)	14 (3)	15 (2)^†^
$P_{\mathrm{ETC}O_{2}}$ (mmHg)	37.5 (2.2)	37.2 (1.9)	37.1 (1.3)	33.7 (1.3)^*^	31.2 (1.4)^#*^	30.5 (1.4)^†*^
SAP (mmHg)	129 (3)	121 (5)^#^	122 (6)^†^	130 (5)	124 (10)^#^	125 (7)^†^
DAP (mmHg)	77 (3)	71 (4)^#^	74 (5)	78 (3)	74 (3)^#^	75 (3)

Significant difference # between CWI_A_ and CWI_B_, † between CWI_A_ and CWI_C_, ‡ between CWI_B_ and CWI_C_, and * between normoxic and hypoxic trial (*P* ≤ 0.05). CWI_L‐60_, the last 60 min of each cold‐water immersion; DAP, diastolic arterial pressures; EE, energy expenditure; *f*_R_, respiratory frequency; $P_{\mathrm{ETC}O_{2}}$, partial pressure of end‐tidal carbon dioxide; RER, respiratory exchange ratio;

SAP, systolic arterial pressures; ${\dot{V}}_{CO_{2}}$ carbon dioxide production; *V*_T_, tidal volume; ${\dot{V}}_{E}$, expired ventilation; ${\dot{V}}_{O_{2}}$, oxygen uptake; %Shiv_peak_, estimated peak shivering metabolic rate.

${\dot{V}}_{E}$ and *V*_T_ were consistently higher in hypoxia than in normoxia (*P* ≤ 0.01; Table 1). In hypoxia, ${\dot{V}}_{E}$was enhanced gradually over the repeated CWIs; it was higher in CWI_B_ than in CWI_A_ (*P* < 0.001), and in CWI_C_ than in CWI_A_ and CWI_B_ (*P* < 0.01). In hypoxia, *f*_R_ was greater in CWI_C_ than in CWI_A_ (*P *= 0.001; Table 1). $P_{\mathrm{ETC}O_{2}}$ was always lower in hypoxia than in normoxia (*P* < 0.001), and was lower in the hypoxic CWI_B_ and CWI_C_ than the hypoxic CWI_A_ (*P *= 0.001; Table 1). RER dropped in all CWIs (*P* < 0.001); regardless of the breathing condition, it was lower in CWI_B_ and CWI_C_ than in CWI_A_ (*P* < 0.01; Table 1).

**FIGURE 4:**
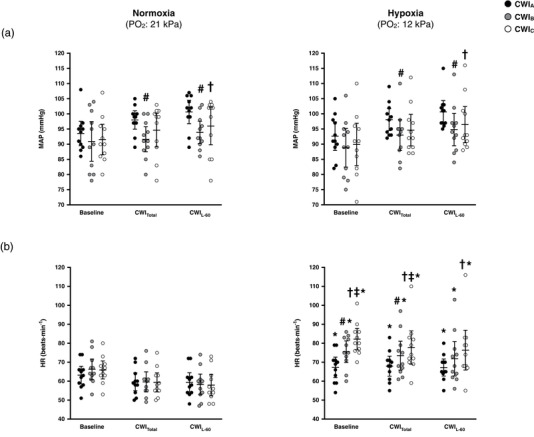
Mean (95% CI) and individual values of mean arterial pressure (MAP; a), and heart rate (HR; b) obtained during the baseline, the total (CWI_Total_), and the last 60 min (CWI_L‐60_) period of the three successive cold‐water (20°C) immersions (CWI_A_: 1st trial, CWI_B_: 2nd trial, CWI_C_: 3rd trial) performed in normoxia and hypoxia. Data in all trials were significantly different from baseline (*P* ≤ 0.02). Significant difference # between CWI_A_ and CWI_B_, † between CWI_A_ and CWI_C_, ‡ between CWI_B_ and CWI_C_, and * between normoxia and hypoxia

In both breathing conditions, the cold‐induced elevation in MAP was blunted in CWI_B_ and CWI_C_ (*P* ≤ 0.05). HR was consistently higher in hypoxia than in normoxia (*P* < 0.001). In hypoxia, HR was higher in CWI_B_ than in CWI_A_, and in CWI_C_ than in CWI_A_ and CWI_B_ (*P* < 0.01; Figure 4b).

**FIGURE 5:**
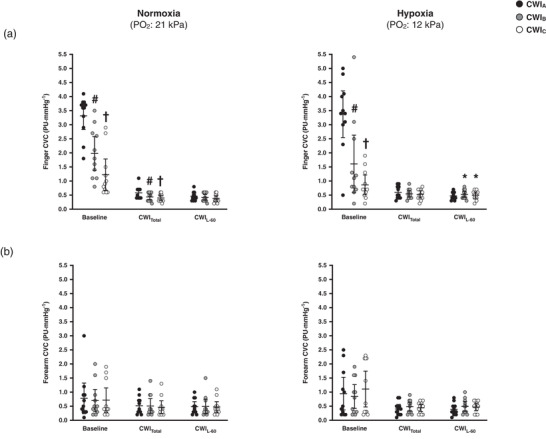
Mean (95% CI) and individual values of cutaneous vascular conductance (CVC) of the left index finger (a) and forearm (b) during the baseline, the total (CWI_Total_), and the last 60 min (CWI_L‐60_) period of the three successive cold‐water (20°C) immersions (CWI_A_: 1st trial, CWI_B_: 2nd trial, CWI_C_: 3rd trial) performed in normoxia and hypoxia. Data in all trials were significantly different from baseline (*P* ≤ 0.01). Significant difference # between CWI_A_ and CWI_B_, † between CWI_A_ and CWI_C_, and * between normoxia and hypoxia. PU, perfusion unit

The baseline finger CVC was lower in CWI_B_ and CWI_C_, regardless of the breathing condition (*P* = 0.01; Figure 5a). In normoxia, the cold‐induced reduction in finger CVC was greater in CWI_B_ and CWI_C_ than in CWI_A_ (*P* ≤ 0.05). Finger CVC did not differ between the three hypoxic CWIs. At CWI_L‐60_, finger CVC was greater in the hypoxic CWI_B_ and CWI_C_ than in the normoxic CWI_B_ and CWI_C_, respectively (*P* ≤ 0.02). Neither hypoxia nor the repeated CWIs affected forearm CVC (Figure 5b).

The mean values of radial, ulnar and brachial arterial flow are presented in Table 2. During the normoxic baseline phases, the flow was lower in the radial and ulnar arteries in CWI_B_ and CWI_C_ than in CWI_A_ (*P* ≤ 0.01); a similar tendency was observed in the brachial artery, but the difference was not statistically significant (*P* = 0.10). During the hypoxic baseline phases, the flow in the three arteries was lower in CWI_B_ and CWI_C_ than in CWI_A_ (*P* < 0.001). The ulnar artery flow was higher in the hypoxic than the normoxic CWI_A_ baseline (*P* = 0.05). In all three arteries, the cold‐induced drop in flow was similar across all CWIs.

**TABLE 2:** Absolute and relative (% changes from baseline) values of blood flow in the radial, ulnar and brachial arteries during the three successive cold‐water (20°C) immersions (CWI_A_: 1st trial, CWI_B_: 2nd trial, CWI_C_: 3rd trial) performed in normoxia and hypoxia

	Normoxia ($P_{O_{2}}$: 21 kPa)						Hypoxia ($P_{O_{2}}$: 12 kPa)					
	CWI_A_		CWI_B_		CWI_C_		CWI_A_		CWI_B_		CWI_C_	
	ml min^−1^	%	ml min^−1^	%	ml min^−1^	%	ml min^−1^	%	ml min^−1^	%	ml min^−1^	%
Radial artery flow												
Baseline	36.8 (10.8)		22.7 (8.9)^#^		18.6 (8.7)^†^		39.9 (15.2)		21.5 (7.3)^#^		21.0 (7.2)^†^	
30 min	8.7 (1.7)	−71 (11)	9.8 (2.0)	−47 (19)^#^	8.2 (1.3)	−39 (25)^†^	12.3 (6.8)	−67 (11)	10.7 (2.3)	−44 (15)^#^	9.3 (2.6)	−52 (15)^†^
60 min	7.5 (1.3)	−75 (9)	7.6 (1.6)	−57 (18)^#^	7.4 (1.4)	−44 (24)^†^	8.0 (2.9)	−74 (14)	7.3 (1.7)	−60 (12)	8.0 (2.9)	−60 (9)^†*^
Final	6.6 (1.2)	−78 (10)	6.8 (1.3)	−61 (18)^#^	6.0 (1.0)	−56 (16)^†^	7.4 (2.8)	−77 (11)	7.9 (2.5)	−54 (22)^#^	6.8 (2.1)	−64 (11)
Ulnar artery flow												
Baseline	47.4 (16.8)		25.7 (8.0)^#^		24.3 (6.1)^†^		59.5 (11.0)^*^		31.4 (13.7)^#^		25.3 (12.3)^†^	
30 min	12.3 (4.5)	−64 (19)	13.2 (4.8)	−35 (28)^#^	10.0 (2.3)	−54 (12)^‡^	12.4 (3.6)	−78 (7)	12.7 (6.6)	−42 (32)^#^	10.3 (3.1)	−47 (18)^†^
60 min	7.8 (2.5)	−77 (14)	10.4 (3.7)	−49 (21)^#^	8.9 (3.0)	−58 (15)^†^	10.1 (2.7)	−82 (5)	8.5 (2.6)	−64 (13)^#^	9.1 (2.0)	−52 (15)^†^
Final	7.2 (1.8)	−78 (12)	7.7 (2.2)	−64 (11)	7.8 (1.7)	−64 (11)	7.6 (1.9)	−87 (3)	8.2 (2.2)	−59 (23)^#^	8.0 (1.6)	−58 (13)^†^
Brachial artery flow												
Baseline	133.9 (29.2)		113.6 (36.9)		105.1 (31.9)		151.6 (19.2)		95.8 (21.6)^#^		83.1 (20.1)^†^	
30 min	66.2 (9.6)	−46 (14)	64.7 (16.5)	−39 (12)	66.7 (17.9)	−34 (11)	70.4 (11.7)	−53 (7)	74.5 (16.2)	−19 (18)^#*^	67.1 (14.5)	−16 (14)^†^
60 min	58.1 (10.9)	−52 (15)	56.9 (9.9)	−43 (16)	57.2 (13.7)	−41 (13)	60.2 (13.9)	−60 (9)	60.6 (8.5)	−33 (12)^#^	58.3 (7.5)	−24 (15)^†^
Final	54.2 (9.3)	−57 (8)	59.9 (11.0)	−41 (13)	52.2 (6.7)	−44% (12)	60.3 (7.5)	−59 (6)	68.2 (13.2)	−19 (31)^#*^	57.8 (8.2)	−24 (19)^†^

Values are mean (95% CI). Data in all trials were significantly different from baseline (*P* ≤ 0.001). Significant difference # between CWI_A_ and CWI_B_, † between CWI_A_ and CWI_C_, ‡ between CWI_B_ and CWI_C_, and * between normoxia and hypoxia (*P* ≤ 0.05).

### 3.3 Perceptual responses

**FIGURE 6:**
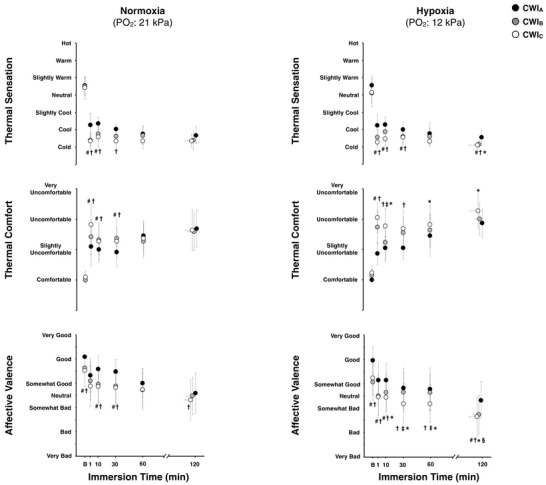
Mean (95% CI) values of thermal sensation, thermal comfort and affective valence during the three successive cold‐water (20°C) immersions (CWI_A_: 1st trial, CWI_B_: 2nd trial, CWI_C_: 3rd trial) performed in normoxia and hypoxia. Data are expressed as a function of the immersion time completed by all subjects, including the final value obtained in each CWI. B: baseline. Data in all trials were significantly different from baseline (*P* ≤ 0.001). Significant difference # between CWI_A_ and CWI_B_, † between CWI_A_ and CWI_C_, ‡ between CWI_B_ and CWI_C_, § between the normoxic and hypoxic CWI_B_, and * between the normoxic and hypoxic CWI_C_

The mean time series for thermal sensation, thermal comfort and affective valence are presented in Figure 6. Regardless of the breathing condition, subjects felt colder and thermally more uncomfortable during the initial part of CWI_B_ and CWI_C_ than the corresponding part of CWI_A_ (*P* < 0.05). Yet hypoxia aggravated the sensation of coldness (*P* = 0.05) and thermal discomfort (*P* = 0.04) at the end of CWI_C_. Overall, the rates of affective valence were lower in the CWI_B_ and CWI_C_ baseline than in the CWI_A_ baseline (*P* ≤ 0.04). In normoxia, subjects felt less pleasant during the first 30 min of CWI_B_ and CWI_C_ than during the corresponding part in CWI_A_, and at the end of CWI_C_ (*P* ≤ 0.04). In hypoxia, the feeling of displeasure was compounded by the last two CWIs, especially by the CWI_C_ (*P* ≤ 0.05).

The mean values of the Profile of Mood States‐Short Form subscales are summarized in Table 3. In both breathing conditions, vigour was impaired, and fatigue was enhanced during CWI_B_ and CWI_C_ (*P* ≤ 0.05); hypoxia aggravated the sensation of fatigue (*P* ≤ 0.05). Moreover, in hypoxia, the self‐reported tension and confusion were higher in CWI_B_ and CWI_C_ than in CWI_A_ (*P* ≤ 0.05), and the perceived depression was enhanced in CWI_C_ compared to in CWI_A_ and CWI_B_ (*P* = 0.05).

**TABLE 3:** Mean (95% CI) values of the Profiles of Mood States‐Short Form (POMS‐SF) subscales before (B_A_: 1st baseline, B_B_: 2nd baseline, B_C_: 3rd baseline) and during the three successive cold‐water (20°C) immersions (CWI_A_: 1st trial, CWI_B_: 2nd trial, CWI_C_: 3rd trial) performed in normoxia and hypoxia

	Normoxia ($P_{O_{2}}$: 21 kPa)						Hypoxia ($P_{O_{2}}$: 12 kPa)					
	B_A_	CWI_A_	B_B_	CWI_B_	B_C_	CWI_C_	B_A_	CWI_A_	B_B_	CWI_B_	B_C_	CWI_C_
Tension	2.4 (1.4)	2.4 (2.5)	1.6 (1.9)	1.7 (1.3)	0.7 (1.6)	1.6 (1.8)	1.9 (1.7)	3.5 (2.3)	2.2 (2.4)^*^	3.4 (2.5)^*^	1.5 (2.0)^†^	3.7 (3.8)^*^
Depression	0.5 (1.0)	0.6 (0.9)	0.4 (0.5)	0.4 (0.5)	0	0.5 (1.2)	0.6 (0.9)	1.5 (1.3)	0.6 (1.2)	1.0 (1.8)	0.9 (1.3)	2.3 (3.6)^‡*^
Vigour	8.4 (3.4)	6.9 (2.7)	5.6 (2.5)^#^	5.0 (2.9)^#^	5.0 (2.9)^†^	5.0 (3.2)^†^	8.8 (3.4)	7.1 (3.4)	4.6 (2.9)^#^	4.9 (2.9)^#^	3.8 (3.6)^†^	4.1 (3.8)^†^
Anger	0.2 (0.3)	1.3 (1.8)	0.7 (1.2)	0.5 (0.6)	0.4 (0.6)	1.0 (1.8)	0.7 (1.0)	1.1 (1.2)	0.9 (1.6)	1.2 (2.0)	1.1 (1.4)	2.5 (3.7)
Confusion	0.5 (0.6)	1.5 (1.3)	1.1 (0.9)	0.7 (0.7)	0.5 (0.6)	1.1 (1.3)	0.5 (0.6)	2.2 (1.5)	1.7 (1.5)^#^	2.0 (1.6)^*^	1.4 (1.4)^†^	2.6 (2.4)^*^
Fatigue	1.1 (0.9)	2.2 (1.7)	3.5 (1.7)^#^	3.2 (1.9)^#^	4.1 (2.6)^†^	4.7 (2.9)^†^	1.8 (1.4)	2.7 (1.0)	6.0 (2.0)^#*^	4.3 (2.4)^#^	7.8 (3.3)^†*^	8.4 (4.2)^†‡*^

Cronbach's α coefficient: tension, 0.95; depression, 0.87; vigour, 0.95; anger, 0.92; confusion, 0.93; fatigue, 0.90. Significant difference # between CWI_A_ and CWI_B_, † between CWI_A_ and CWI_C_, ‡ between CWI_B_ and CWI_C_, and * between normoxic and hypoxic trial (*P* ≤ 0.05).

## 4 DISCUSSION

The study sought to determine, in male lowlanders, the impact of hypoxia on thermoregulatory functions during prolonged intermittent cold stress. We therefore employed a repeated‐measures design, wherein subjects were exposed, for a ∼10 h period, to either normoxia or normobaric hypoxia, while a 2 h fixed moderate‐intensity cold stimulus (i.e. passive immersion in 20°C water) was applied sequentially at 2 h intervals. To eliminate any potential confounding influences on thermoeffector activity, the time of the day during which the serial immersions were performed was the same in the two test sessions, and subjects’ pre‐immersion *T*_rec_, body posture, as well as energy and fluid intakes were similar across all trials.

The present results support previous evidence (Castellani et al., 1998) that, in normoxia, repeated whole‐body cold provocations subjected in a single day may increase susceptibility to develop hypothermia. Also, in line with other human studies (Cipriano & Goldman, 1975; Johnston et al., 1996; Keramidas et al., 2019; Robinson & Haymes, 1990), acute systemic hypoxia (i.e. in CWI_A_) aggravated the cold‐induced drop in body core temperature. However, and contrary to our hypothesis, the ∼10 h sustained exposure to hypoxia mediated a gradual upregulation of endogenous heat production, which appeared to prevent the progressive hypothermic response prompted by the serial cold stimuli. Moreover, the hypoxic stressor progressively degraded mood, and compounded the perceived thermal discomfort, and sensations of fatigue and coldness.

### 4.1 Cold‐defence effector responses to repeated cold stress

The normoxic CWI_C_ augmented the cold‐induced fall in *T*_rec_, a finding that seems to be in accord with a previous work using an experimental design similar to the present (Castellani et al., 1998). In that study (Castellani et al., 1998), the hypothermic response induced by the repeated CWIs was attributed to a centrally mediated alteration in the recruitment of shivering‐engaged muscles, given the delay in shivering onset. In the present study, however, a modest elevation in $\dot{M}$ was manifest during the initial part (i.e. until the 70th minute) of the normoxic CWI_C_ (Figure 2a). On the basis of a previous work (Castellani, Young, Kain, & Sawka, 1999), it is highly unlikely that such shift in cold‐induced $\dot{M}$ was related to its circadian variation. Although the origin of the increased $\dot{M}$ cannot be identified, we speculate that it might represent the enhanced recruitment of non‐shivering thermogenic processes, involving mainly the skeletal muscle (e.g. the mitochondrial uncoupling, the calcium cycling), and perhaps to a minute extent brown adipose tissue thermogenesis (see Haman & Blondin, 2017; van Marken Lichtenbelt & Schrauwen, 2011; Wijers, Schrauwen, Saris, & van Marken Lichtenbelt, 2008). It might also reflect an overall increase in tonic motor unit activity evoking heat‐producing isometric contractions (i.e. thermoregulatory muscle tone), which typically precede the onset of the overt tremorlike movements of shivering (Burton & Bronk, 1937; Lømo, Eken, Bekkestad Rein, & Nja, 2019; Meigal et al., 2003). Regardless of its source, this slight elevation in endogenous heat production, was insufficient to defend the body core temperature. Notably, during the later portion of CWI_C_, the thermal drive for metabolic heat generation appeared to be blunted: the lower *T*_rec_ failed to augment thermogenesis. A similar metabolic desensitization has been described during prolonged continuous cold‐water immersion (Tikuisis, 2003; Tikuisis et al., 2002).

Judging by the absolute values obtained during the immersions, neither *T*_sk_ (Figure 1b) nor the blood flow in the non‐immersed cutaneous (Figure 5) and muscle (Table 2) vasculatures was substantially affected by the repeated normoxic CWIs. It might be argued, however, that the peripheral vasomotor reactivity to cold was somewhat impaired, since the relative changes (i.e. the magnitude of the cold‐induced drop from the respective baseline values) in *T*_sk_, finger CVC, and radial and ulnar arterial flow (Table 2) were diminished. In addition, the MAP response to cold stimulus was blunted during the repeated CWIs (Figure 4a). Attenuation of the pressor response has also been induced by sustained periods of exertional fatigue (e.g. after military sustained operations; Keramidas, Gadefors, Nilsson, & Eiken, 2018a; Young et al., 1998), as well as by short‐ and long‐term regimens of cold adaptation (Keramidas, Kolegard, & Eiken, 2018b; Makinen et al., 2008; O'Brien et al., 2000; Tipton et al., 2013), and has been attributed primarily to a peripheral adrenergic desensitization (Opstad, 1990). Collectively, and regardless of the underlying thermoregulatory mechanisms, current results further indicate that, in normoxia, perturbations of thermal homeostasis imposed by repetitive whole‐body exposures to moderate cold may enhance susceptibility to hypothermia.

Acute hypoxia aggravated the reduction in deep body temperature during CWI_A_, a result that conforms to those from previous investigations (Cipriano & Goldman, 1975; Gautier, Bonora, Schultz, & Remmers, 1987; Johnston et al., 1996; Keramidas et al., 2019; Robinson & Haymes, 1990). The novel finding of this study was that the hypoxic impact on cold thermoregulation appeared to be time dependent. The sustained exposure to hypoxia obviated the progressive hypothermic response prompted by the serial cold stimuli; given that, contrary to in normoxia, *T*_rec_ did not differ between the hypoxic CWI_A_ and CWI_C_ (Figure 1a). Interestingly, unlike the transient hypometabolism commonly observed in hypoxic animals (Dzal & Milsom, 2019; Tattersall & Milsom, 2009), the ∼10 h hypoxic stressor augmented, in a gradual manner, the cold‐induced thermogenesis. This seemingly paradoxical increase in metabolic thermosensitivity to the fixed 20°C water stimulus was independent of the thermal inputs from the body core (*T*_rec_ was similar between the hypoxic trials) and the shell (*T*_sk_ was slightly elevated during CWI_C_; see next paragraph). The response was probably mediated by distinct acts of hypoxia on central neural circuits that initiate and regulate thermogenesis (Morrison & Nakamura, 2019); the precise region of action, however, cannot be determined by the current experimental design. We speculate that an enhancement in sympathoadrenergic activity, typically occurring during sustained hypoxic exposure (Kanstrup et al., 1999; Saito et al., 1988), although MAP was not enhanced in this study, might have exerted an influence on the thermoregulatory centres, modifying the integrative control function, and subsequently sensitizing the heat‐producing thermoeffector. Moreover, it has been shown that, in anesthetized cats, the hypoxic stressor directly increases the activity of thermosensitive neurons, including the cold‐responsive neurons, in the preoptic area of the anterior hypothalamus (Tamaki & Nakayama, 1987). Also, the metabolic cost associated with the hypoxia‐evoked hyperpnoea and tachycardia might have contributed, at least to some extent, to the enhanced $\dot{M}$. That hypoxia stimulated the thermogenic response by exciting other inputs to the hypothalamus appears less plausible. For instance, the hypoxia‐evoked stimulation of peripheral chemoreceptors (Mott, 1963), and the development of hypocapnia (Gautier et al., 1987) would have been expected to suppress shivering. Likewise, baroreceptor stimulation may augment shivering in anaesthetized rabbits (Ishii & Ishii, 1960), whereas a reduction in MAP response was noted in the present hypoxic CWI_B_ and CWI_C_.

In line with previous work (Cipriano & Goldman, 1975; Johnston et al., 1996; Keramidas et al., 2019; Simmons et al., 2010), systemic hypoxia attenuated the magnitude of cold‐induced peripheral vasoconstriction, which was attended by a small (∼0.4°C), but significant, elevation in *T*_sk_ during the CWI_L‐60_ of CWI_C_. The present results thus indicate that sustained hypoxia may modulate the cold‐defence thermoeffectors in a divergent manner: the hypoxic stressor facilitated thermogenesis, whereas concurrently it partly compromised heat conservation. Still, the robust metabolic response was sufficient to compensate for the increased rate of heat loss, and hence to prevent a potentiation of the hypothermia during the course of the serial cold stimuli. Whether the hypoxic influence on peripheral vasculature was determined by central or peripheral mechanisms is unclear. The dissimilar effects of hypoxia on peripheral vasoreactivity and thermogenesis are consistent with the notion that separate integrators and pathways probably exist for each thermal sensor‐to‐effector loop (McAllen, Tanaka, Ootsuka, & McKinley, 2010; Taylor & Gordon, 2019).

### 4.2 Thermoperceptual responses to repeated cold stress

The thermal perception, which in turn initiates conscious thermo‐behavioural actions, is driven primarily by thermoafferent inputs from the body core and the shell (Schlader & Vargas, 2019). Although their relative contribution to the thermoperceptual output is still unclear, a hierarchical arrangement seems to exist, whereby internal body temperature constitutes the main determinant, especially for thermal (un)pleasantness (Attia, 1984; Mower, 1976). During the first 30 min of the normoxic CWI_B_ and CWI_C_, subjects’ ratings for coldness and thermal discomfort were amplified. The sensory and hedonic evaluation of the 20°C water stimulus appeared to be dissociated from central and peripheral thermal cues; *T*_rec_ and *T*_sk_ were similar in the three normoxic trials. This transient enhancement in thermal discomfort was probably associated with subjects’ psychological state prior to and during the initial part of the immersion. In particular, the preceding CWI(s) in the normoxic test session provoked a gradual shift towards a less pleasant generalized affective state, which in turn might have influenced the central integration of temperature information (Arnsten, 2015), enhancing thermoperceptual responsiveness (Auliciems, 1981; Barwood, Corbett, Tipton, Wagstaff, & Massey, 2017). It is noteworthy, however, that after the first 30 min of immersion, any inter‐trial difference in thermoperception dissipated. During the normoxic CWI_C_, the lower values in Δ*T*_rec_, and the somewhat greater generalized unpleasantness, appeared to be inadequate stimuli to invoke distinct temperature‐related sensations. Presumably, the serial cold‐water immersions might have elicited a habituation effect (Golden & Tipton, 1988; O'Brien et al., 2000), which did emerge when the impact of psychological strain on thermoperception was attenuated.

In line with previous studies (Golja & Mekjavic, 2003; Golja et al., 2005; Keramidas et al., 2019), acute hypoxia (i.e. CWI_A_) influenced neither the discriminative nor the hedonic perceptions to cold stimulus. The sensation of coldness and thermal displeasure, however, were exacerbated by the hypoxic CWI_B_ and CWI_C_, a response that prevailed throughout the cold provocations. This thermoperceptual sensitization was independent of the magnitude of cold‐induced drop in *T*_rec_, which did not vary between the trials, and in *T*_sk_, which was either similar, or even slightly diminished in the latter part of CWI_C_. The aggravated perceptual responses to cold can probably be ascribed to hypoxia *per se*, and its direct impact on neural circuits mediating thermal sensation and comfort (Craig, 2002); yet our study does not allow us to draw any firm conclusions on the underlying neural mechanisms. It is also plausible that the feelings of coldness and thermal discomfort were dictated by the negative affective state and the enhanced levels of perceived fatigue produced by hypoxia. Lastly, the conscious perception of the augmented shivering in hypoxia could have provoked, independently, some degree of discomfort (Gagge, Stolwijk, & Hardy, 1967), which, conceivably, might have magnified the thermal discomfort (Schlader & Vargas, 2019). Whether the magnitude of thermoperceptual sensitization induced by hypoxia would eventually facilitate the decision‐making to thermoregulate behaviourally remains to be settled.

### 4.3 Acute mountain sickness and thermoregulatory function

Based on the Lake Louise Score values, significant acute mountain sickness occurred in seven subjects. Symptoms were accentuated during the second half of the test session (≥4 h in hypoxia), and mainly included headache and light‐headedness; two subjects complained of nausea as well. It is highly unlikely that the latter symptom was ascribed to hypoxia‐induced malabsorption of food, given that no evidence exists that this occurs at altitude lower than 5500 m (Kayser, Acheson, Decombaz, Fern, & Cerretelli, 1992; Mekjavic et al., 2016). The blood glucose availability was also maintained, despite the moderate calorie intake during the 11 h session. The impact of acute mountain sickness on human thermoregulation remains perplexing. Field studies (Maggiorini, Bartsch, & Oelz, 1997; Roggla, Moser, Wagner, & Roggla, 2000) have suggested that increases in body core temperature might be associated with the development of acute mountain sickness, whereas Loeppky et al. (2003) have demonstrated that, during a 12 h hypoxic confinement in a hypobaric chamber, a reduction in internal temperature was related to acute mountain sickness. In the present study, no correlation was detected between the Lake Louise Score values and the cold‐induced Δ*T*_rec_ (*r*_s_ = 0.12; *P* = 0.59), or $\dot{M}$ (*r*_s_ = 0.32; *P* = 0.14). Still, whether, or to what extent, acute mountain sickness‐depended perturbations in autonomic function might have contributed to the augmented metabolic and thermoperceptual responsiveness to cold is unclear.

### 4.4 Methodological limitations and delimitations

Considering that the magnitude of stress reactivity is determined by the intensity, duration and mode of (inter‐)action of stressor(s) encountered, the findings of our study are limited to the methodological constraints employed. Thus, it remains to be settled whether the responses observed herein would be similar in: (i) hypobaric hypoxic circumstances, (ii) moderate degrees of hypoxia, and (iii) prolonged continuous and/or severe cold stress. Furthermore, whether the current results are applicable to cold‐air conditions, which represent a more realistic scenario at high altitude, remains unknown. An inter‐individual variation in the magnitude and direction of the thermoeffector responses was also observed. Yet, given the small sample size and the relatively homogeneous group of subjects tested (as regards their age, sex, aerobic capacity and body morphology), the source of the detected variation cannot be identified (see Atkinson & Batterham, 2015). Further work is also required to determine whether a sex‐specific influence of hypoxia on cold thermoregulation exists, provided that the thermoregulatory function might differ in women, due to morphological differences and/or the hormonal fluctuation during the menstrual cycle (Burse, 1979).

Subjects were sufficiently rewarmed prior to each CWI, as indicated by the baseline *T*_rec_ values. Yet the rewarming treatments may have eliminated the circadian fluctuations in body internal temperature (Krauchi & Wirz‐Justice, 1994), thus marginally attenuating the natural afternoon elevation in *T*_rec_. Also, whether the rate of body‐core cooling was confounded by the slightly lower baseline *T*_sk_ noted prior to the afternoon CWIs (CWI_A_ *vs*. CWI_B_: ∼0.5°C; CWI_A_ *vs*. CWI_C_: ∼0.8°C) is unclear. The changes in body core temperature were evaluated based on *T*_rec_, which is regarded a slow indicator and might be influenced by variations in rectal (mucosal) and abdominal blood flow (see Taylor, Tipton, & Kenny, 2014). In addition, the study would have benefited from the integration of a multimodal quantification of shivering, in particular by the use of surface electromyography (Arnold et al., 2020; Haman & Blondin, 2017).

Arguably, any changes in body fluid distribution and plasma volume typically evoked by CWI (Stocks et al., 2004) and sustained hypoxia (Sawka, Convertino, Eichner, Schnieder, & Young, 2000) might have influenced, at least partly, our findings; despite that the immersion depths as well as subjects’ body postures did not vary between the trials. However, the similar drop in subjects’ body weight observed at the end of each test session suggests that the magnitude of body fluid loss during a session was probably similar in hypoxia and normoxia.

Both in normoxia and hypoxia, the steep increase in $\dot{M}$ observed during the initial 10 min of CWIs was attributed to hyperventilation, which, along with tachycardia, describe the main components of the ‘cold‐shock response’ (Tipton, 1989). In line with previous work (Barwood et al., 2017), the magnitude of hyperventilation tended to be blunted by the three repeated normoxic CWIs, especially by CWI_C_ (changes from the baseline in the normoxic CWI_A_, 126 (46)%; CWI_B_, 105 (48)%; CWI_C_, 87 (26)%; *P* = 0.10), whereas this was not the case in hypoxia (changes from the baseline in the hypoxic CWI_A_, 99 (39)%; CWI_B_, 118 (71)%; CWI_C_, 123 (28)%; *P* = 0.55). Yet, in the present study, subjects immersed themselves into the water tank, and the initial ventilatory responses might have been confounded by other non‐thermal influences (e.g. excessive body movements), and hence these data should be interpreted with caution.

To evaluate the efficiency of the blinding process, subjects were asked, in the middle of each test session, to report whether they believed they were breathing a normoxic or a hypoxic gas. Subjects replied 22 times, of which they were indecisive nine times, guessed incorrectly four times, and correctly nine times. It cannot be excluded, however, that the thermoperceptual responses might have been biased, at least to some extent, by subjects’ awareness of the breathing condition (i.e. a placebo effect). Lastly, the use of a unipolar scale for thermal (dis)comfort might have dampened any variation between the breathing conditions, especially during the pre‐immersion phase.

### 4.5 Practical perspectives – does hypoxia counteract ‘shivering fatigue’ or impede metabolic habituation to cold?

Castellani et al. (1998) have postulated that, in normoxia, protracted intermittent cold stress leads to a *forced* reduction in body core temperature, possibly reflecting a condition of ‘thermoregulatory fatigue’. On the basis of this premise, the present results might suggest that prolonged hypoxia prevents the development of ‘shivering fatigue’, hence reducing the risk of accidental hypothermia. Such a notion must, however, be questioned. Although no direct assessment of fatigue was conducted in this study, a large body of evidence has documented that diminished systemic O_2_ availability does in fact facilitate both central (i.e. a reduction in central motor drive) and peripheral (i.e. functional changes at or distal to the neuromuscular junction) fatigue (Goodall, Twomey, & Amann, 2014).

Rather, our findings may suggest that the repeated cold stress elicited a *regulated* hypothermic response (i.e. habituation), which ostensibly was disturbed by the hypoxic stressor. Assuming that the metabolic habituation to cold confers a survival advantage by preserving energy reserves and gross motor function (Carlson, Burns, Holmes, & Webb, 1953; Golden & Tipton, 1988), the excessive metabolic response instigated by hypoxia may provide an adaptive benefit in short‐term (as observed herein), but might in fact be disadvantageous for long‐term survival in a moderate cold environment. In addition, the concurrent activation of opposing thermoeffectors (i.e. enhancement of both heat‐producing and heat‐dissipating responses) observed in hypoxia could also be an energy‐costly process over time. Altogether, these hypoxia‐driven adjustments might describe a thermoregulatory ‘allostatic overload’ (McEwen, 2007; Ramsay & Woods, 2014); this concept, however, remains, hypothetical, and warrants further investigation. Finally, it is also likely that, in real field conditions, any thermoregulatory benefits obtained from the hypoxia‐induced enhancement in $\dot{M}$ would be overridden by a more prominent acute mountain sickness, which is often prevalent during prolonged (i.e. ≥4–6 h) and moderate‐to‐severe (i.e. ≥2500 m) altitude exposure.

In conclusion, the present findings support previous evidence that, in male lowlanders, the cold‐induced drop in deep body temperature may be aggravated by (i) repeated moderate cold stress encountered within a single day (Castellani et al., 1998), and (ii) acute systemic hypoxia (Cipriano & Goldman, 1975; Johnston et al., 1996; Keramidas et al., 2019; Robinson & Haymes, 1990). A ∼10 h sustained exposure to hypoxia, however, appears to mediate metabolic and thermoperceptual sensitization to whole‐body cold stress, and hence to prevent the progressive hypothermic response prompted by the serial cold stimuli. The nature and function of such a hypoxia‐dependent adaptive response should be elucidated in future studies.

## COMPETING INTERESTS

None declared.

## AUTHOR CONTRIBUTIONS

The study was performed in a laboratory of the Division of Environmental Physiology, KTH‐Royal Institute of Technology (Solna, Sweden). M.E.K. and O.E. conception and design of research; M.E.K. and R.K. performed experiments; M.E.K. and R.K. analysed data; M.E.K. and O.E. interpreted results of experiments; M.E.K. prepared figures; M.E.K. drafted the article; M.E.K., R.K. and O.E. edited and revised the article. All authors approved the final version of the article, and agree to be accountable for all aspects of the work in ensuring that questions related to the accuracy or integrity of any part of the work are appropriately investigated and resolved. All persons designated as authors qualify for authorship, and all those who qualify for authorship are listed.

## Data Availability

The data that support the findings of this study are available from the corresponding author upon reasonable request.
